# Reactive Pad-Steam Dyeing of Cotton Fabric Modified with Cationic P(St-BA-VBT) Nanospheres

**DOI:** 10.3390/polym10060564

**Published:** 2018-05-23

**Authors:** Kuanjun Fang, Dawu Shu, Xiuming Liu, Yuqing Cai, Fangfang An, Xinqing Zhang

**Affiliations:** 1 School of Textiles, Tianjin Polytechnic University, No. 399 Binshui Xi Road, Xiqing District, Tianjin 300387, China; shudawu@126.com (D.S.); yushimylove@163.com (X.L.); 13933186174@163.com (F.A.); jeanking5056@163.com (X.Z.); 2 Collaborative Innovation Center for Eco-Textiles of Shandong Province, No. 308 Ningxia Road, Qingdao 266071, China; 3 Key Laboratory of Science & Technology of Eco-Textile, Ministry of Education, Donghua University, Shanghai 201620, China; 4 School of Textiles and Clothing, Qingdao University, No. 308 Ningxia Road, Qingdao 266071, China; 13954216129@163.com

**Keywords:** reactive dye, cotton, cationic nanospheres, pad-steam dyeing, fixation

## Abstract

The Poly[Styrene-Butyl acrylate-(P-vinylbenzyl trimethyl ammonium chloride)] P(St-BA-VBT) nanospheres with N^+^(CH_3_)_3_ functional groups were successfully prepared and applied to modify cotton fabrics using a pad-dry process. The obtained cationic cotton fabrics were dyed with pad-steam dyeing with reactive dye. The results show that the appropriate concentration of nanospheres was 4 g/L. The sodium carbonate of 25 g/L and steaming time of 3 min were suitable for dyeing cationic cotton with 25 g/L of C.I. Reactive Blue 222. The color strength and dye fixation rates of dyed cationic cotton fabrics increased by 39.4% and 14.3% compared with untreated fabrics. Moreover, sodium carbonate and steaming time were reduced by 37.5% and 40%, respectively. The rubbing and washing fastness of dyed fabrics were equal or higher 3 and 4–5 grades, respectively. Scanning electron microscopy (SEM) images revealed that the P(St-BA-VBT) nanospheres randomly distributed and did not form a continuous film on the cationic cotton fiber surfaces. The X-ray photoelectron spectroscopy (XPS) analysis further demonstrated the presence of cationic nanospheres on the fiber surfaces. The cationic modification did not affect the breaking strength of cotton fabrics.

## 1. Introduction

Cotton is one of the most widely used natural polymer fibers in textiles and is usually dyed with reactive dyes [1,2]. For reactive dyeing of cotton, electrostatic interaction between the dye and the fiber mainly determine the final color performance [3]. Large quantities of inorganic salts (NaCl or Na_2_SO_4_) need to be added into dye bath to enhance the utilization of reactive dyes [4]. The discharge of colored wastewater with high salinity leads to serious water pollution and land salinization [5,6]. However, grafting cotton with cationic compounds is an effective approach for increasing the dye–fiber substantivity [7] and avoiding the use of inorganic salts [5,8].

Various kinds of cationic compounds have been investigated for the cationic modification of cotton [9,10,11]. 3-chloro-2-hydroxypropyl trimethylammonium chloride (CHPTAC) is the most preferred cationic agent for the cationization of cotton fabrics [10,12,13]. A nicotinoyl thioglycolate (NTG) was used to increase the dye reactivity and reduce the salt use in the dyeing process [14]. Cotton fabrics grafted with 2,4,6-tri[(2-hydroxy-3-trimethyl-ammonium)propyl]-1,3,5-triazine chloride and 2,4,-bichloro[(6-sulfanilic acid)-1,3,5-triazine] had satisfactory dyeing properties using salt-free dyeing process [15]. However, these cationic agents have poor stability, and easily result in uneven dyeing [16]. In contrast to the linear compounds with low molecular weight, polymer compounds with a large number of reactive groups are of high stability, and the cotton fabrics grafted with these compounds could achieve excellent dyeing performance in absence of inorganic salts [7,17]. For example, the cotton fabrics modified with amino-terminated hyper-branched polymer (HBP-NH_2_) showed dramatically enhanced color depth [18]. By introducing 8 and 64 amino ends groups to the dendrimers, the modified fabrics could be dyed with anionic dyes under salt-free dyeing conditions [11]. The poly(ethyleneimine) [19], cationic starches [20], and chitosanpoly(propyleneimine) dendrimer hybrid [21] were also successfully grafted on cotton fiber surfaces to obtain the dark colors without using salts. Although appreciable progress has been made for enhancing dye utilization by using these functional polymers, they still have the drawbacks of the poor air permeability and handle of dyed fabrics. In recent years, cationic nanospheres with large specific surface areas and positive charges had been used for fiber modification to realize the acid dye and pigment dyeing of cotton fabrics [16,22]. However, it has been rarely reported the pad-steam dyeing of cotton fabrics modified with cationic nanospheres using reactive dyes.

In this study, cationic P(St-BA-VBT) nanospheres with N^+^(CH_3_)_3_ functional groups were synthesized and grafted on cotton fabrics using a pad-dry process, and then the fabrics were dyed with C.I. Reactive Blue 222 (Blue 222) in a pad-steam dyeing process. The effects of nanospheres concentration, alkali amount, steaming time and dye concentration on dyeing properties of cotton fabrics were investigated. The surface morphology and chemical composition of cotton fabrics were analyzed by SEM and XPS, respectively. The fastness and breaking strength of cotton fabrics were also evaluated. The results show that the cationic fabrics had the merits of low chemical consumption, short steaming time, high dye utilization and outstanding color build-up property.

## 2. Materials and Methods

### 2.1. Materials

**Figure 1:**
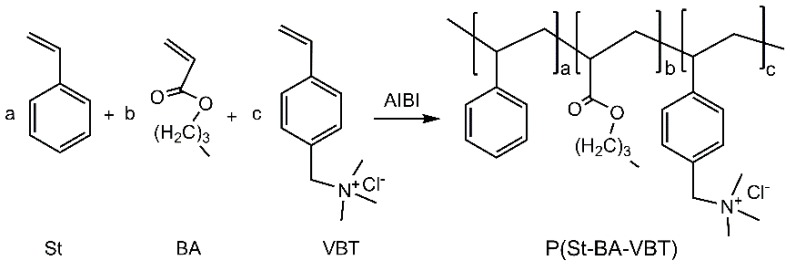
The polymerization for preparing the cationic P(St-BA-VBT) nanospheres.

The commercially desized, scoured and bleached plain weave cotton fabrics of 176 g/m^2^ were supplied by Sunvim Group Co., Ltd., Gaomi, China. Analytical grade of sodium carbonate (Na_2_CO_3_) was obtained from Tianjin Kemiou Chemical Reagent Co., Ltd., Tianjin, China. Styrene (St) was purchased from Tianjin Ruijinte Chemical Reagent Co., Ltd., Tianjin, China. Butyl acrylate (BA) was provided by Tianjin BASF Chemical Co., Ltd., Tianjin, China. P-vinylbenzyl trimethyl ammonium chloride (VBT) was purchased from Tianjin Heowns Biochem Technologies (Tianjin, China). The cationic P(St-BA-VBT) nanospheres were prepared according to Guo et al. [23] and the polymerization for preparing the cationic nanospheres was illustrated in Figure 1. C.I. Reactive Blue 222 containing monochlorotriazine and vinylsulfone reactive groups was kindly supplied by ANOKY group Co., Ltd., Shanghai, China.

### 2.2. Grafting Cationic Nanospheres on Cotton Fabrics

The cotton fabrics were firstly immersed in the modification solutions containing 1–5 g/L of cationic P (St-BA-VBT) nanospheres and 0.1 g/L of Na_2_CO_3_ for 5 min. Then, the wet fabric samples were passed through a padding mangle (PO-B, Laizhou Yuanmao Instrument, Co., Ltd., Laizhou, China) to obtain (70 ± 1)% fabric pickup using one-dip, one-nip procedure. Subsequently, the padded samples were immediately dried at 80 °C for 6 min in a DGG-101-2BS drying oven (Tianjin Tianyu Experimentation Instrument Co. Ltd., Tianjin, China) to obtain the cationic cotton fabrics.

### 2.3. Reactive Pad-Steam Dyeing

A 100-mL Blue 222 solution was mixed with 25 mL Na_2_CO_3_ solution to make up the padding liquor. The padding liquor was stirred for 1 min in 250 mL beaker and transferred into a groove of the padder. Cotton fabrics were subjected to the padding liquors to obtain (70 ± 1)% pick-up using the padder with the one-dip, one-nip technique at room temperature. Then, the padded samples were rapidly steamed for 0.5–7.0 min to fix the reactive dyes in a saturated steam atmosphere. The sample was divided into two parts; one was dried at room temperature as the steamed sample. The other was rinsed with running tap water, and washed with hot water (50–60 °C) for 3 min. The soaping-off was done at 100 °C for 15 min in the presence of 3 g/L standard soap flakes. Finally, the soaped samples were rinsed with 70–80 °C water for 3 min and thoroughly washed with running tap water. In the washing process, the liquor ratio was set as 50:1. These fabrics were dried at room temperature and called as the washed samples.

### 2.4. Characterization

#### 2.4.1. Nanospheres Properties

The hydrodynamic particle size of cationic P(St-BA-VBT) nanospheres was determined by a dynamic light scattering instrument (Malvern Nano-Zs90 nano-particle size analyzer, Malvern Worcestershire, UK). The Zeta potential was measured by a Zeta potential analyzer (Malvern, Malvern Worcestershire, UK). All certain amount of P(St-BA-VBT) nanospheres samples were diluted 2000-fold by deionized water before test. The glass transition temperature of P(St-BA-VBT) nanospheres was characterized by a differential scanning calorimetry (Netzsch DSC 204F1, Netzsch Group, Bavaria, Germany) within the temperature ranged from 20 to 200 °C [24].

#### 2.4.2. Colorimetric Data

The colorimetric data of dyed fabrics were performed using a Datacolor SF-600 plus (Datacolor Co., Lawrenceville, NJ, USA) with D_65_ illumination and 10° standard observer [25]. The color strength (*K*/*S*) was assessed at the maximum absorption wavelength of 620 nm. Each fabric was folded to four layers and measured at ten different locations, and the average *K*/*S* value was calculated based on the Kubelka–Munk Equation as shown in Equation (1):

(1) $$K/S={\left(1-R\right)}^{2}/2R\;$$

where *R* is reflectance of the dyed fabric at the maximum absorption wavelength, *K* and *S* are spectral absorbance and scattering coefficients of dyed fabrics, respectively.

#### 2.4.3. Dye Fixation Rate

Dye fixation rate was measured according to GB/T 27592-2011 (Reactive dyes-determination of degree of fixation in pad dyeing). The dyed cotton fabrics were firstly cut into fine pieces. Then, 0.1 g (accurate to 0.0001 g) of the fine samples were weighed and dissolved with 10 mL 75% H_2_SO_4_ solution in a 100-mL volumetric flask at room temperature. After the fabric samples were completely dissolved, the solutions were diluted to 100 mL with deionized water and shaken well for testing. The solution absorbance was measured at the maximum absorbance wavelength of the dye solution using an UV-3200 Spectrophotometer (Shanghai Mapada Instruments Co., Ltd., Shanghai, China). And the fixation rate of each dyed fabric was determined by using the Equation (2) [26]:

(2) $$F=\frac{A_{2}/m_{2}}{A_{1}/m_{1}}\times 100\%$$

where *F* is the fixation rate of reactive dye (%), *A*_1_ and *A*_2_ are the absorbance of the diluted sulfuric acid solutions of the steamed and washed samples, respectively. *m*_1_ is the dry weight of the steamed sample and *m*_2_ is the dry weight of the washed sample.

#### 2.4.4. Surface Morphology Observation by SEM

The cotton fabric was firstly immersed the modification solution (containing 4 g/L of nanospheres and 0.1 g/L of Na_2_CO_3_) and then passed through a padding mangle to obtain 70% pickup. The padded sample were dried at 80 °C for 6 min. The obtained fabric was hereinafter referred to as cationic cotton fabric. The surface morphology of the cationic and untreated fabrics was observed using S-4800 field scanning electron microscope (Hitachi, Tokyo, Japan) operating at 10 kV. All samples were coated with gold before the observation.

#### 2.4.5. XPS Analysis

The chemical compositions of both the cationic and untreated cotton fabrics were analyzed using a K-Alpha X-ray photoelectron spectrometer (Thermo Fisher Scientific Co., Ltd., Waltham, MA, USA) with an Al K-Alpha source type at an incident energy of 1486.6 eV. For analyzing the general spectra, the spot size, pass energy and energy step size were separately set as 400 µm, 200.0 eV and 1.0 eV. All measurements were made at an UHV chamber pressure between 5 × 10^−9^ and 2 × 10^−8^ Torr. The C1s peak was referred to a C–C binding energy of 285.0 eV [27].

#### 2.4.6. Rubbing and Washing Fastness

Dry and wet rubbing fastness properties were measured according to ISO 105-X12: 2001 using an Y571 rubbing machine (Nantong Hongda Experiment Instruments Co., Ltd., Nantong, China). The washing fastness was tested according to the methods established in ISO 105-C10:2007 using an SW-24 washing colorfastness tester (Laizhou Yuanmao Instrument, Co., Ltd., Laizhou, China).

#### 2.4.7. Breaking Strength

The breaking strength of cotton fabrics was tested by the YG065 electronic fabric strength tester (Laizhou Electron Instrument Co., Ltd., Laizhou, China) according to the method of GB/T 3923.1-2013: Textiles-tensile properties of fabrics-part 1. The effective length and width of the fabrics were 250 and 50 mm, respectively. The tensile velocity was set as 100 mm/min.

## 3. Results and Discussion

### 3.1. Properties of Cationic P(St-BA-VBT) Nanospheres

The particle size and Zeta potential of cationic P(St-BA-VBT) nanospheres were 63 nm and +56.9 mV, respectively. The results indicated that the cationic nanospheres had high surface area and positive reaction sites. The glass transition temperature of P(St-BA-VBT) nanospheres was 95.7 °C, meaning that the nanospheres could be used in the modification and dyeing process of cotton fabrics.

### 3.2. Effect of the Nanospheres on Pad-Steam Dyeing of Cotton

**Figure 2:**
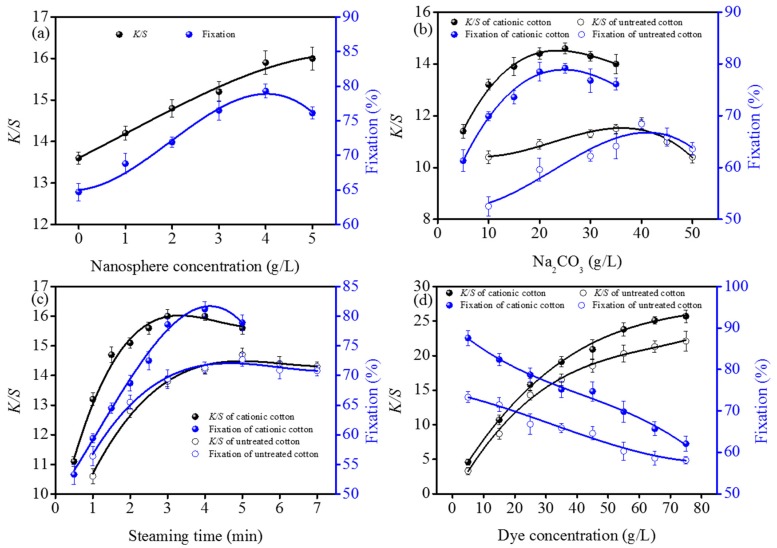
Effect of cationic nanosphere concentrations: (**a**) Na_2_CO_3_ amounts, (**b**) steaming time, (**c**) and dye concentration (**d**) on the *K*/*S* values and dye fixation rates of dyed cotton fabrics. All the pickup of fabrics were (70 ± 1)% and steaming temperature was (102 ± 1) °C.

The dyeing properties of cotton fabrics were determined by the nanosphere concentrations, alkali amounts, steaming time and dye concentration [28]. In order to evaluate the dyeing properties of cotton fabrics grafted with the nanospheres, the dyeing conditions were optimized based on the dye fixation rates and *K*/*S* values of cotton fabrics dyed with Blue 222 (As shown in Figure 2).

#### 3.2.1. Nanosphere Concentrations

Figure 2a shows the effect of nanosphere concentrations on the fixation rates and *K*/*S* values of dyed cotton fabrics with Blue 222. The compositions of dye solution was 25 g/L of Blue 222 and 30 g/L of Na_2_CO_3_, the padded fabrics were steamed at 102 °C for 3 min. As the concentration of cationic nanospheres was increased from 0 to 4 g/L, the *K*/*S* values and fixation rates of dyed fabrics respectively increased from 13.6 to 15.9 and form 66.5% to 79.3%, indicating the higher the nanosphere concentration, the darker the color and the higher the dye fixation rates of dyed fabrics. This is because the cationic reaction sites on cotton fabrics increased as the nanosphere concentration increased. However, further increasing the cationic nanosphere concentration to 5 g/L resulted in a slow growing of *K*/*S* value and decrease of dye fixation rate. This could be explained by that the considerable amount of dyes fixed on the outmost accessible area of fibers through covalent bond and electrostatic attraction. Thereafter the 4 g/L of nanospheres was selected in following investigation.

#### 3.2.2. Alkali Amounts

The effect of Na_2_CO_3_ amounts on *K*/*S* values and fixation rates of cotton fabrics dyed with 25 g/L of Blue 222 was illustrated in Figure 2b. When Na_2_CO_3_ concentration was increased from 5 to 25 g/L, the *K*/*S* values and fixation rates of dyed cationic fabrics increased by 28.1% and 17.9%, respectively. Further increasing the Na_2_CO_3_ from 25 to 35 g/L resulted in a slow downtrend of both *K*/*S* value and dye fixation rate. It is also clear that the influence of Na_2_CO_3_ concentration on the *K*/*S* values and dye fixation rates of untreated cotton is similar to those of cationic cotton fabrics, indicating that the insufficient or excessive amount of Na_2_CO_3_ results in the reduction of dye fixation rates. This phenomenon could be explained from the perspective of competing among various reactions: the activation of dye reactive groups and cellulose hydroxyl [29], the covalent bonds between reactive dye and fibers as well as dye hydrolysis [30,31]. The maximum *K*/*S* value (11.7) and dye fixation rate (68.4%) of untreated cotton fabrics were obtained at 40 g/L of Na_2_CO_3_. As expected, the *K*/*S* values and dye fixation rates of dyed cationic fabrics were significantly higher than those of untreated cotton fabrics at the same Na_2_CO_3_ concentration. Meanwhile, the optimal Na_2_CO_3_ concentration of cationic cotton (25 g/L) was lower than that of untreated cotton (40 g/L). The reason is that some reactive dyes could be spontaneously adsorbed on the cationic cotton fibers through electrostatic attraction [24], leading to the higher dye fixation rates and the lower Na_2_CO_3_ consumption compared with the untreated cotton fabrics.

#### 3.2.3. Steaming Times

Figure 2c shows the variations of *K*/*S* values and dye fixation rates with the steaming time. To determine the steaming time, Blue 222 concentration was 25 g/L, the Na_2_CO_3_ concentration was 25 and 40 g/L for the cationic and untreated cotton fabrics, respectively. It is clear that the *K*/*S* value and dye fixation rate of dyed cationic cotton increased with extending the steaming time. When steaming time increased to 3 min, the *K*/*S* value reached the maximum value of 16.0. At 4 min the fixation rate increased to the maximum value of 81.2%. Furthering prolonging the steaming time, both the *K*/*S* values and the dye fixation rates decreased. In the pad-steam dyeing process, the dye molecules could be quickly absorbed on the surfaces of cationic cotton fibers via electrostatic attraction, resulting that the maximum *K*/*S* values was obtained at a short steaming time. The maximum fixation rate reached at a longer steaming time because the Blue 222 molecules need more time to diffuse into the fiber interior and to react with the cellulosic hydroxyl anions. For the untreated cotton fabric, both the maximum *K*/*S* value (14.7) and the maximum fixation rate (72.7%) obtained at 5 min, which is longer than the cationic cotton. It is also obvious that the *K*/*S* values and dye fixation rates of dyed cationic cotton fabrics were always higher than those of untreated cotton fabrics at the same steaming time, indicating that the cationic cotton obtained darker color and higher dye utilization than the untreated cotton. This phenomenon could be mainly ascribed to electrostatic attraction between the cationic cotton fibers and dye molecules. In terms of saving energy, the optimum steaming time of cationic cotton was 3 min, which is 40% less than that of untreated cotton fabric (5 min).

#### 3.2.4. Dye Concentration

The *K*/*S* values and dye fixation rates of cotton fabrics dyed with the pad-steam dyeing under a dye concentration range of 5 to 75 g/L were illustrated in Figure 2d. Wherein, the Na_2_CO_3_ concentration was 25 and 40 g/L for the cationic and untreated cotton fabrics, respectively. And the steaming time was 3 and 5 min for cationic and untreated cotton fabrics, in accordingly. When the dye concentration increased from 5 to 75 g/L, the *K*/*S* values increased from 4.6 to 25.7 for the cationic fabrics and from 3.3 to 22.1 for the untreated fabrics, whereas the fixation rate decreased from 87.6% to 62.1% and from 73.3% to 58.1%, respectively. It is also clear that the increment of *K*/*S* values was slower when dye concentration was over 55 g/L. As more Blue 222 molecules were fixed on the fibers, there were less available sites for further reactions, leading to a slow increasing trend of *K*/*S* values at higher dye concentration [3]. Compared with untreated cotton fabrics, the *K*/*S* values and dye fixation rates of dyed cationic fabrics were enhanced by 39.4% and 14.3% at the maximum values, respectively. The satisfactory results further indicates that the nanospheres’ modification of cotton fabric provides a novel potential approach to obtain the dark color and high dye fixation rate using reactive pad-steam dyeing based on the combined effect of electrostatic attraction and covalent bonding.

Table 1 shows the color characteristic values of cotton fabrics dyed with different concentration of Blue 222.

It can be seen that the L* values of dyed cationic cotton fabrics are lower than those of untreated cotton fabrics, indicating that the darker color were obtained for the cationic fabrics. a* refers to the redness (+) and greenness (−), b* to the yellowness (+) and blueness (−), C* to the color saturation, and *h°* to the hue [32]. Both the a* and b* of dyed cationic fabrics are negative and the absolute values are lower than those of dyed untreated cotton fabrics, signifying that the color of dyed cationic fabric became less greenness and blueness. The C* values of dyed cationic cotton fabrics are smaller than those of untreated cotton, meaning that the color of the cationic cotton is less bright. The *h°* values of all dyed fabrics are close to 250°, meaning that the color of dyed fabrics mainly appeared Blue due to *h°* = 270° corresponds to a pure blue [33]. The differences of color characteristic values may be ascribed to the different distribution of Blue 222 on the dyed fabrics.

Table 2 shows the rubbing and washing fastness of cotton fabrics dyed with different concentration of Blue 222. As Blue 222 concentration increased, the wet rubbing fastness of dyed fabrics decreased while the washing fastness had no obvious change. All dyed cotton fabrics exhibit excellent rubbing fastness and washing fastness. However, the wet rubbing fastness of cationic cotton fabric dyed with 65 g/L of Blue 222 is lower than that of the untreated fabric. This is because the deeper the shade and the poorer the dye penetration into fiber interior, the lower the wet rubbing fastness [34].

As described above, the cationic cotton fabrics had the advantages of low Na_2_CO_3_ consumption, short steaming time, dark color, as well as high dye fixation rate, which is consistent with what we expected.

### 3.3. Performance Analysis of Cationic and Untreated Fabrics

**Figure 3:**
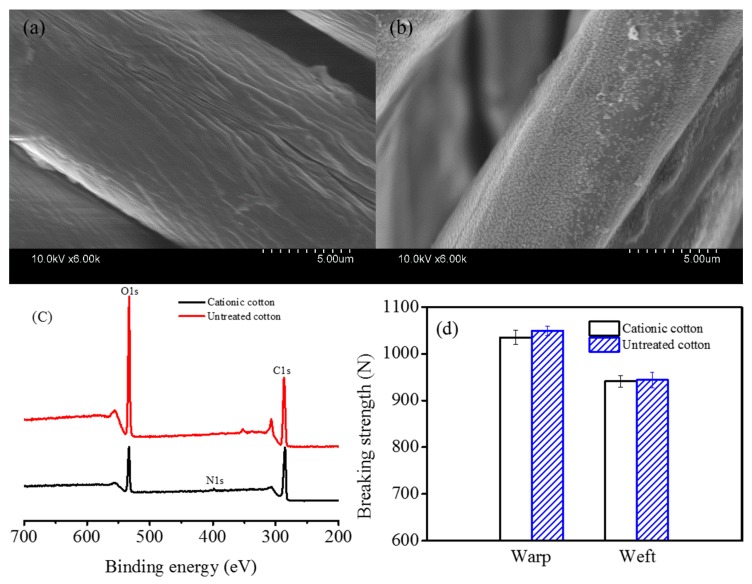
Surface morphology, chemical compositions and breaking strengths of cotton fabrics: (**a**) SEM image of untreated cotton; (**b**) SEM image of cationic cotton; (**c**) XPS Spectra and (**d**) breaking strengths of cotton fabrics.

In order to clearly reveal the properties of cationic cotton and untreated cotton fabrics, the surface morphology, chemical compositions and breaking strength of cotton fabrics were illustrated in Figure 3.

From Figure 3a we can see that the surface of untreated cotton had many cracks and grooves. Although lots of nanospheres deposited on the modified fiber surfaces, the size of nanospheres was obviously smaller than the diameter of cotton fibers, as shown in Figure 3b. Furthermore, the nanospheres were randomly distributed and not formed a continuous film on the surface of cationic cotton fibers, indicating that the cationic modification process had little effect on the diffusion of the dye solutions. Compared with the previously reported [24], the obtained cationic cotton fabrics have the obvious advantages for reactive dyeing.

The general XPS spectra and the elemental content of cotton fabrics are illustrated in Figure 3c and Table 3, respectively. As shown in Figure 3c, the carbon (C1s at 284.8 eV) and oxygen (O1s at 531.2 eV) displayed in the XPS spectrum of untreated cotton fabrics [35]. The appearance of the weak N1s peak at 400.1 eV and the greatly decreased intensity of the O1s peak in the XPS spectrum of the cationic cotton fabric further demonstrates the presence of cationic nanospheres on the surfaces of cationic cotton fabrics.

Table 3 shows that the cationic cotton fabric has lower O/C atomic ratio (0.30) than the untreated cotton fabric (0.64) because the cationic P(St-BA-VBT) nanospheres had a higher carbon component than the cotton fibers. Besides, the nitrogen content increased from 0% to 1.57%, which further verifies that the nanospheres were adsorbed on the fiber surfaces. The cationic monomer (VBT) of the nanospheres contains a quaternary ammonium group N^+^(CH_3_)_3_ (Figure 1) resulting in the increase of the nitrogen content on the cationic cotton fibers. As displayed in Figure 3d, the breaking strength of cationic and untreated cotton fabrics were almost the same, signifying that the modification process does not affect the fabric strength.

### 3.4. Mechanism of Nanospheres Modification and Reactive Dyeing

**Figure 4:**
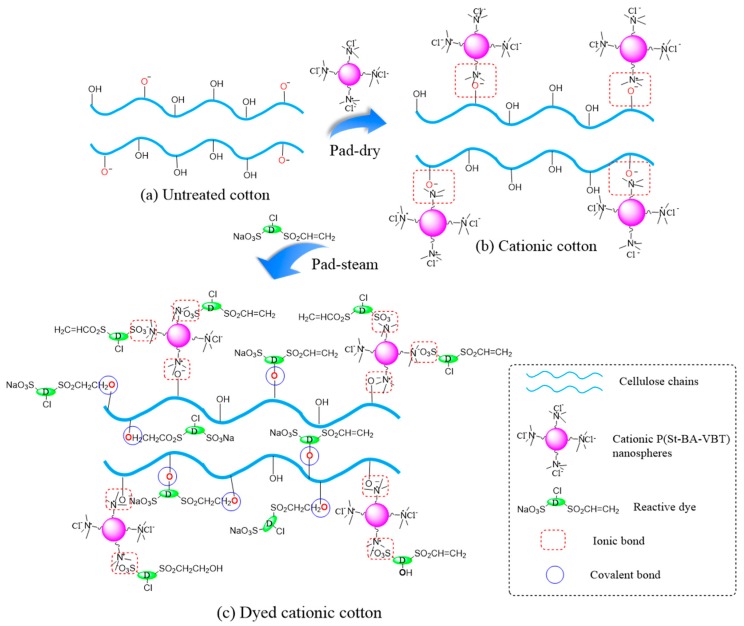
The mechanism of nanospheres modification and reactive dyeing of cotton fabrics: (**a**) untreated cotton; (**b**) cationic cotton and (**c**) dye cationic cotton.

In this work, the cotton fabrics were firstly modified with the cationic P(St-BA-VBT) nanospheres using a pad-dry process to graft the positive charges on fiber surfaces. Then, the cationic cotton fabrics were dyed with Blue 222 in a pad-steam dyeing process. The mechanism of nanospheres modification and reactive pad-steam dyeing is illustrated in Figure 4.

When the cotton fabrics were placed in the alkaline modification solutions, some cellulose hydroxyl groups (Cell–OH) were converted into cellulosate anions (Cell–O^−^) (Figure 4a). The P(St-BA-VBT) nanospheres could be spontaneously adsorbed on the cotton fiber surfaces through the electrostatic attraction [16] between the ammonium salt ions (–N^+^(CH_3_)_3_) and cellulosate anions (Cell–O^−^). As a result, the positive charge bonding sites on the surface of cotton fibers were introduced due to the presence of quaternary ammonium groups (Figure 4b). In the pad-steam dyeing process, the chemical bonds between the reactive dye and the cationic cotton fibers were not only covalent bonds but also ionic bonds (Figure 4c), which decreased the alkali consumption and steaming time, and enhanced the *K*/*S* value and dye fixation rate.

## 4. Conclusions

Cotton fabrics were modified with 4 g/L of cationic P(St-BA-VBT) nanospheres. The sodium carbonate of 25 g/L and steaming time of 3 min were suitable for dyeing cationic cotton with 25 g/L of C.I. Reactive Blue 222. The *K*/*S* values and the dye fixation rates of dyed cationic cotton fabrics increased up to the most 39.4% and 14.3% compared with the untreated cotton fabrics. Moreover, the Na_2_CO_3_ amount and steaming time were reduced by 37.5% and 40%, respectively. The rubbing and washing fastness of dyed cotton fabrics were equal or over 3 and 4–5 grades, respectively. SEM images revealed that the P(St-BA-VBT) nanospheres randomly distributed and did not form a continuous film on the cationic cotton fiber surfaces. The N1s peaks and 1.57% nitrogen content further demonstrated the presence of cationic nanospheres on the fiber surfaces. The breaking strength of cotton fabrics modified with P(St-BA-VBT) nanospheres were almost the same as that of untreated cotton fabrics.

## Figures and Tables

**Table 1:** Color characteristic values of dyed fabrics *^a^*.

Fabrics	Dye Concentration (g/L)	Color Characteristic Values				
		L*	a*	b*	C*	*h*°
Cationic	15	31.3	−9.6	−18.7	21.0	242.9
	35	25.0	−7.1	−16.8	18.2	247.3
	65	20.3	−4.3	−13.2	13.9	252.2
Untreated	15	38.8	−9.9	−19.3	21.7	242.8
	35	28.7	−7.2	−17.5	18.9	247.8
	65	23.4	−5.0	−14.8	15.6	259.3

*^a^* All the pickup of fabrics were (70 ± 1)% and the steaming temperature was (102 ± 1) °C.

**Table 2:** The rubbing and washing fastness of dyed fabrics *^a^*.

Fabrics	Dye Concentration (g/L)	Rubbing Fastness		Washing Fastness		
		Dry	Wet	SC	SW	CC
Cationic	15	4–5	4	5	5	5
	35	4–5	3–4	4–5	4–5	4–5
	65	4–5	3	4–5	4–5	4–5
Untreated	15	4–5	4	5	5	5
	35	4–5	3–4	4–5	4–5	4–5
	65	4–5	3–4	4–5	4–5	4–5

*^a^* Staining on cotton fabric (SC), staining on wool fabric (SW), color change (CC).

**Table 3:** Chemical composition of cationic and untreated cotton fabrics.

Cotton Fabric	Chemical Composition			Atomic Ratio O/C
	C (%)	O (%)	N (%)	
Cationic	74.32	22.58	1.57	0.30
Untreated	57.53	36.72	0	0.64
